# Member announcements

**DOI:** 10.3109/03009730903500363

**Published:** 2009-12-08

**Authors:** Torbjörn Karlsson, Anna Rask-Andersen

Dear Readers

Our society has had a good autumn with many interesting activities, such as our annual meeting on the 17th of September. During the meeting, Björn Zethelius, Lars Berglund, Arvo Hänni and Christian Berne were awarded for the best article published 2008 in Upsala Journal of Medical Sciences. The title of their article was “The interaction between impaired acute insulin response and insulin resistance predict type 2 diabetes and impairment of fasting glucose”. During the meeting we also awarded the two best dissertations from the Faculty of Medicine: Maria Jonsson for her thesis “Use and misuse of oxytocin during delivery” and Göran Ronquist for his thesis “Some characteristics of human prostasomes and their relationship in prostate cancer”.

The honorary lecture was held by Arne Andersson over the subject “Clinical islet transplantation – some still unresolved problems”. The lecture was followed by a wonderful dinner for the members.

The next event was the annual Olof Rudbeck day on the 16th of October, a day jointly arranged by the Upsala Medical Society and the Disciplinary Domain of Medicine and Pharmacy, Uppsala University. This year the theme was vitamin D, and we heard of different aspects of vitamin D in surgery, dermatology, prostatic cancer, the central nervous system, bone and also a lecture on this vitamin, which has become a hormone. This year we had the pleasure to award an Uppsalian scientist with the Olof Rudbeck price. David Bergqvist got the price and he gave a brilliant lecture on his special subject “Aneusrysms- from traumatology to screening”.

The spring to come we are co-arranging a Martin H:son Holmdahl lecture and we will be back with details about where and when.

